# Climate refugia on the Great Barrier Reef fail when global warming exceeds 3°C

**DOI:** 10.1111/gcb.16323

**Published:** 2022-08-02

**Authors:** Jennifer K. McWhorter, Paul R. Halloran, George Roff, William J. Skirving, Peter J. Mumby

**Affiliations:** ^1^ College of Life and Environmental Sciences University of Exeter Exeter UK; ^2^ Marine Spatial Ecology Lab, School of Biological Sciences and ARC Centre of Excellence for Coral Reef Studies University of Queensland St Lucia Queensland Australia; ^3^ Atlantic Oceanographic and Meteorological Laboratory National Oceanic and Atmospheric Administration Miami Florida USA; ^4^ Commonwealth Scientific and Industrial Research Organisation Canberra Australia; ^5^ Coral Reef Watch, National Oceanic and Atmospheric Administration College Park Maryland USA; ^6^ ReefSense Pty Ltd. Townsville Queensland Australia

## Abstract

Increases in the magnitude, frequency, and duration of warm seawater temperatures are causing mass coral mortality events across the globe. Although, even during the most extensive bleaching events, some reefs escape exposure to severe stress, constituting potential refugia. Here, we identify present‐day climate refugia on the Great Barrier Reef (GBR) and project their persistence into the future. To do this, we apply semi‐dynamic downscaling to an ensemble of climate projections released for the IPCC's recent sixth Assessment Report. We find that GBR locations experiencing the least thermal stress over the past 20 years have done so because of their oceanographic circumstance, which implies that longer‐term persistence of climate refugia is feasible. Specifically, tidal and wind mixing of warm water away from the sea surface appears to provide relief from warming. However, on average this relative advantage only persists until global warming exceeds ~3°C.

## Graphical abstract

Increasing global average temperatures are driving mass coral mortality events around the world. Although, there are some reefs that escape the warm exposure, deemed as a potential refugia. We apply a unique method to improve coarse resolution climate projections over the Great Barrier Reef, isolating the locations that have experienced the least amount of thermal stress over the past 20 years. Wind and tidal mixing enabled the persistence of climate refugia in these locations. However, this advantage only persists until global average warming exceeds ~3°C.

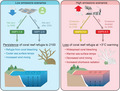

## 1 INTRODUCTION

Tropical corals are one of the most vulnerable groups of organisms to warming temperatures because they live within a narrow thermal threshold (Berkelmans & Willis, 1999; Glynn & D'croz, 1990; Reaser et al., 2000). When anomalously warm temperatures are prolonged and intensified, coral bleaching, a loss of photosynthetic endosymbiotic dinoflagellates (*Symbiodinium* spp.) in the tissue (Berkelmans & Willis, 1999; Glynn & D'croz, 1990; Reaser et al., 2000), can lead to coral mortality (Eakin et al., 2010; Hughes, Anderson, et al., 2018). Global coral bleaching and mortality were initially correlated to warming sea temperatures in the 1982–83 El Niño‐Southern Oscillation (ENSO) event (Glynn, 1984; Robinson, 1985). While historically associated with ENSO events (Baker et al., 2008; Glynn et al., 2001; Kleypas et al., 2015), global‐warming driven increases in sea surface temperatures (SSTs; Bindoff et al., 2013) are decreasing the time between marine heatwaves, escalating the frequency of mass coral mortality events (Hughes, Anderson, et al., 2018; Hughes, Kerry, et al., 2018). These events will continue to become more frequent—reducing recovery time—as greenhouse gas emissions continue (McWhorter et al., 2021).

The large‐scale geographical pattern of global warming is defined by more rapid warming at the poles than at lower latitudes (Holland & Bitz, 2003), particularly in the Northern Hemisphere (Cohen et al., 2014) and amplification of warming over land in contrast with the oceans (Byrne & O'gorman, 2013). Warm air moving off the land can warm shallow coastal seas, which have been warming faster than deeper waters (Heron et al., 2016) owing to their lower heat capacity. However, tropical coastal oceans experience a more complex pattern of change (Liao et al., 2015), with the superposition of natural variability associated with climate modes such as ENSO on top of these global trends. Despite the significance of ENSO, the relative stability of low‐latitude climates means their temperatures are emerging from the variability they have historically experienced faster than elsewhere on the planet (Hawkins et al., 2020), a factor critical to coral bleaching (Safaie et al., 2018). From 1985 to 2012 tropical ocean warming was most rapid in the Indian Ocean and slowest in the Atlantic Ocean, a pattern seen also in bleaching season temperatures (Heron et al., 2016). More recently, the Pacific appears to be experiencing the most extreme and frequent heating events (Skirving et al., 2019), leading to high coral mortality (Eakin et al., 2019).

Regional‐scale mass coral bleaching events have increased in frequency and severity since the early 1980s (Beyer et al., 2018; Darling et al., 2019; Hughes, Anderson, et al., 2018; Skirving et al., 2019). In a global study of bleaching intensity between 1980 and 2016, Hughes, Anderson, et al. (2018) found that the most recurrent and highest intensity bleaching occurred in the Western Atlantic affecting >50% of locations prior to 2010. This was followed by the Pacific Ocean where warming events increased after 2010 (Hughes, Anderson, et al., 2018). Record oceanic and atmospheric temperatures then drove the largest global‐scale coral bleaching event that lasted from 2014 to 2017 (Eakin et al., 2019; Skirving et al., 2019). This event resulted in a rapid decline of coral reefs worldwide (Eakin et al., 2019; Skirving et al., 2019). However, even during the most extensive bleaching events, areas have been observed that have consistently not bleached (Baird et al., 2018; Eakin et al., 2019).

‘Climate refugia’ in the context of exposure to climate stress have been defined as areas where low frequency or severity of bleaching conditions are expected to persist longer into the future than surrounding areas (Chollett & Mumby, 2013; Dixon et al., 2022; Glynn, 1996; Kavousi & Keppel, 2018; Morelli et al., 2016; Riegl & Piller, 2003; Van Hooidonk et al., 2013, 2016). Numerous studies have developed methods for quantifying refugia in terms of ecosystem vulnerability. In these studies, ecosystem vulnerability is considered a response to thermal exposure, species‐specific resistance, and capacity for recovery (Beyer et al., 2018; Bozec et al., 2020; Cheung et al., 2021; Hock et al., 2017; Kavousi & Keppel, 2018; Morelli et al., 2016).

Small scale ecological differences on the reef can determine the heterogeneous impacts of thermal exposure. Coral morphology and physiology influence the responses of endosymbionts and the animal to bleaching (Dunn et al., 2012; Fitt et al., 2009; Grottoli et al., 2006, 2014; Loya et al., 2001; Rodrigues & Grottoli, 2007; Wilkinson & Hodgson, 1999) with changes in coral assemblage and function often following a warming event (Hughes, Anderson, et al., 2018; Loya et al., 2001; Marshall & Baird, 2000; Stimson et al., 2002).

Bleaching conditions over individual coral reefs can be linked to weather conditions such as reduced cloud cover, higher than normal air temperature, and higher than normal atmospheric pressure conditions (Gonzalez‐Espinosa & Donner, 2021; McGowan & Theobald, 2017). Clouds have been shown to provide shading during warming events by limiting the amount of shortwave radiation and reducing coral heat stress (Gonzalez‐Espinosa & Donner, 2021; McGowan & Theobald, 2017; Mumby et al., 2001). In fact, the changing light conditions from winter to summer has been shown to provide a cumulative effect on light stress experienced by corals, and therefore modulating the effect of temperature (Skirving et al., 2017). Low wind speeds and neap tides, observed during the 1998 Great Barrier Reef bleaching event (Skirving & Guinotte, 2000) result in reduced mixing of heat away from the surface water and lower turbidity. Increased suspended particulate load, associated with turbidity, can reduce the penetration of shortwave radiation into the water column, providing shading and short‐term relief from bleaching as identified by Cacciapaglia and van Woesik (2016). In addition to wind and tidal mixing, mesoscale processes such as boundary currents and eddies can provide thermal relief (Chollett & Mumby, 2013; Glynn, 1996), while also supplying the reef with larvae (Hock et al., 2017) and food (Grottoli et al., 2006).

The conditions through which refugia arise are highly localized. This presents a challenge when trying to explore their behaviour or project their future state using models. Global climate models (GCMs) typically have a coarse horizontal resolution of around 1° and are unable to resolve important mesoscale features in the coastal zone (Van Hooidonk et al., 2016). The course resolution means that shallow coastal waters are rarely accounted for in models, and consequently processes such as tidal mixing are not simulated. Without representing the hydrodynamics occurring in shelf seas, GCMs are unable to simulate the heat stress experienced by tropical coral reefs adequately (Donner et al., 2005; Kwiatkowski et al., 2013). Here, we use an ensemble of semi‐dynamic downscaled climate models to examine the locations of refugia in the context of thermal exposure and their persistence under a range of climate projections on the GBR as a case study for identifying refugia worldwide.

### 1.1 Study design

To improve the resolution of climate projections in the coastal environment one can use dynamic or statistical downscaling (Halloran et al., 2021; Van Hooidonk et al., 2013, 2015, 2016). Here, we apply a semi‐dynamical downscaling approach (see Section 4 for detailed information), utilising the S2P3‐R v2.0 model (Halloran et al., 2021), to projections from the newly released 6th phase of Coupled Model Intercomparison Project (CMIP6) (O'Neill et al., 2016). We consider three shared socio‐economic pathways (SSP), SSP1, SSP3, and SSP5 (Riahi et al., 2017) and four emission trajectories (SSP1‐1.9, SSP1‐2.6, SSP3‐7.0, SSP5‐8.5) (Riahi et al., 2017) explored across five climate models, MRI‐ESM2‐0 (Adachi et al., 2013), EC‐Earth3‐Veg (Döscher et al., 2021), UKESM1‐0‐LL (Sellar et al., 2019), CNRM‐ESM2‐1 (Séférian et al., 2019), IPSL‐ESM2‐0 (Boucher et al., 2020). These models were selected based on the availability of atmospheric variables: surface atmosphere air temperature, winds, air pressure, humidity, and net longwave and shortwave radiation, at the initial release of CMIP6 data (April 2021). Our downscaling approach simulates the detailed temperature response resulting from the interaction of the CMIP6 models' meteorology with local tides and bathymetry. The model domain spans 142.0° W, 157.0° E, 30.0° S, 10.0° S from 4 to 50 m water depth, at a 10 km horizontal resolution and 2 m vertical resolution.

Downscaled SST was used to derive standard metrics of coral thermal stress; The National Oceanic and Atmospheric Administration (NOAA) Coral Reef Watch's degree heating weeks (DHW) (Donner et al., 2005; Skirving et al., 2020). DHW refers to a measurement of anomalous warm temperatures accumulating over a summer, or 3‐month period. DHW correlates strongly with coral bleaching/mortality, even though the nature of such relationships change as more susceptible corals are lost through bleaching (Hughes et al., 2017; Hughes, Kerry, et al., 2018). To find present‐day locations of less impacted reefs in the context of climate exposure (refugia), we identified the locations with the lowest 20% of averaged DHW values from 1999 to 2019 from climate model simulations, in line with previous approaches (Cheung et al., 2021; Hock et al., 2017). These locations are used throughout the study to determine spatial patterns of global warming on the GBR.

## 2 RESULTS

### 2.1 Climate refugia

**FIGURE 1:**
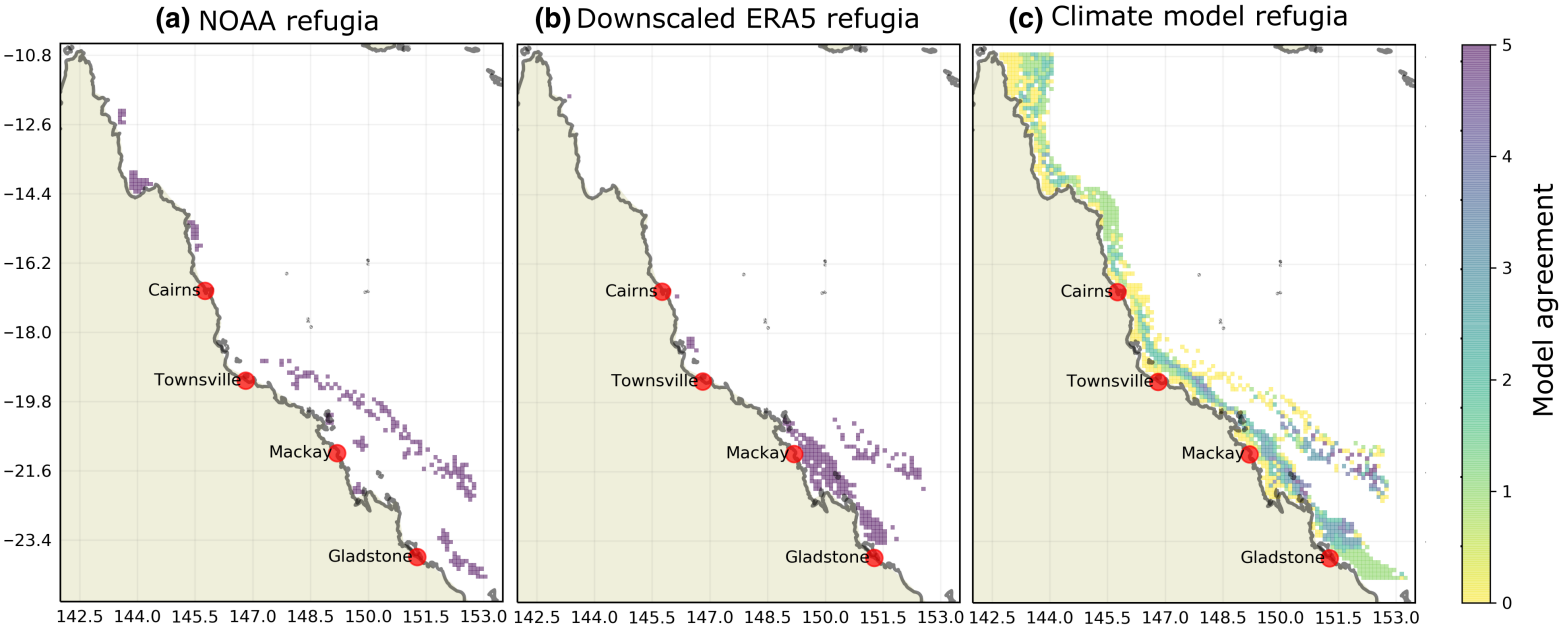
Spatial agreement is shown through observation‐based products and model outputs. (a) Refugia locations derived from the National Oceanic and Atmospheric Administration (NOAA) Coral Reef Watch degree heating weeks (DHWs) product, 1999–2019. (b) Refugia locations from the downscaled ERA5 atmospheric product. (c) Climate model agreement on refugia locations from five downscaled CMIP6 models, MRI‐ESM2‐0, EC‐Earth3‐Veg, UKESM1‐0‐LL, CNRM‐ESM2‐1, IPSL‐CM6A‐LR. The 0 value, or yellow, represents areas of no agreement from any models. The 5 value, or dark purple, represents the most agreement, from all 5 models.

Using satellite‐derived observations of DHW (Donner et al., 2005; Skirving et al., 2020), we found evidence of refugia around the Swains (near 21° S, the widest component of the GBR, onshore to offshore) as well as offshore Mackay and offshore Gladstone and the east coast of the Cape York Peninsula (Figure 1a). We define refugia locations as being the lowest 20% of climatological DHWs (see Section 4). We then confirmed that such observations were broadly consistent with the downscaled climate model output, based on the atmospheric conditions experienced over the same 20‐year interval (1999–2019) as used for the refugia calculation (Figure 1b). Despite climate model simulations being only a plausible realisation of the weather, our CMIP6 driven results identified a consistent geographical pattern to the refugia locations across models and with observations (Figure 1c). Such agreement between mechanistic models and observations strongly implies that these refugia occur because of fundamental local oceanographic or meteorological attributes, rather than by chance.

**FIGURE 2:**
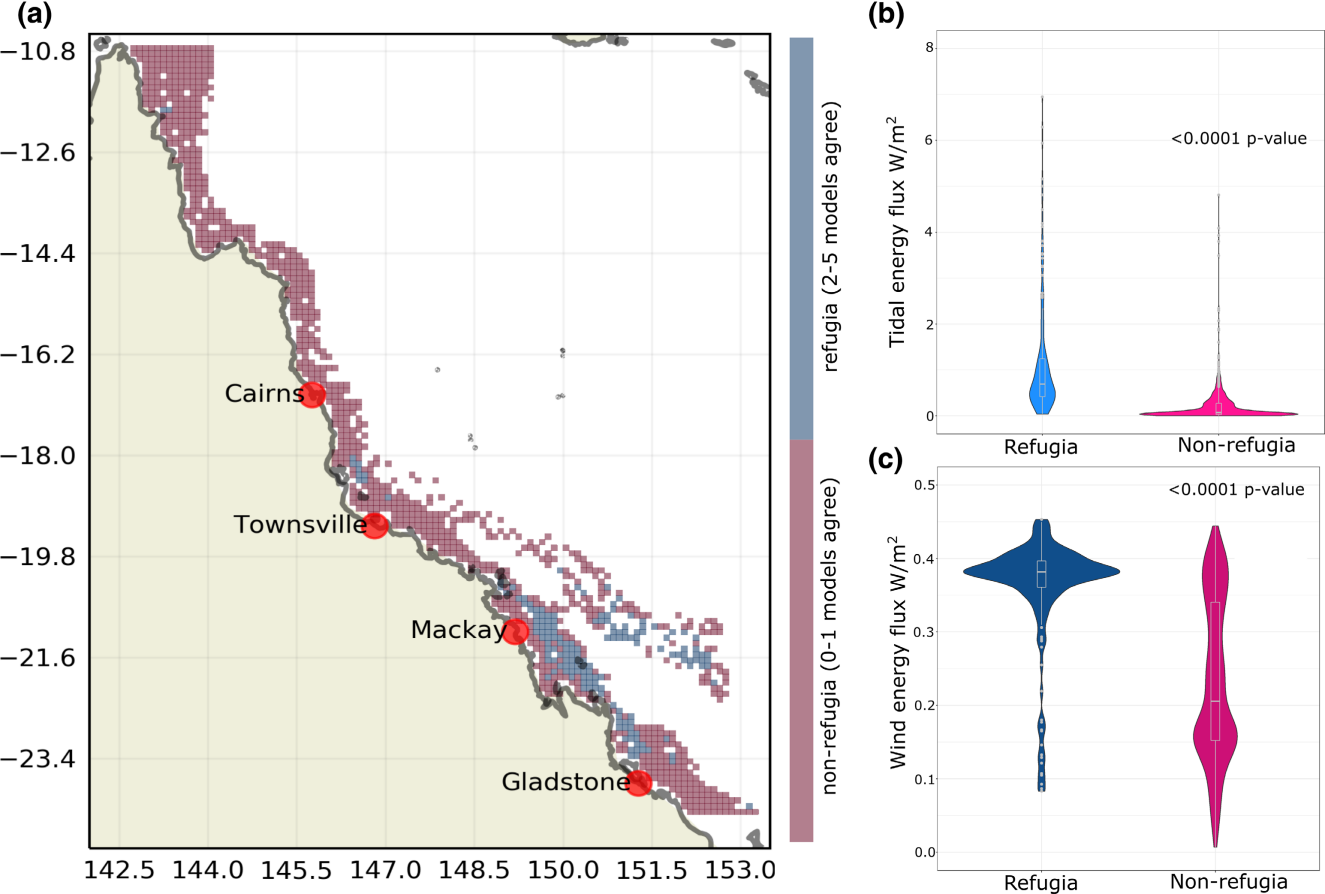
The downscaled outputs of the atmospheric reanalysis product ERA5 were used to further validate climate model agreement of refugia and non‐refugia locations and test for tidal and wind energy in refugia and non‐refugia locations. (a) The map shows where the climate refugia from two or more downscaled climate models agree with the downscaled ERA5 refugia locations from 1999 to 2019. (b) Tidal and (c) wind energy are shown for refugia and non‐refugia locations as violin plots to display the probability density and significance testing using the emmeans package in R. Wind energy calculations are based on the mean of these austral summer bleaching years, 2002, 2016, 2017, and summer months, December, January, February, March. (b) Not included are outliers reaching up to 18 in the tidal energy, the y‐axis was limited between 0.8 to better display most data.

Exploring the mechanisms leading to refugia using the downscaled ERA5 observational product for the last 20 years, we find that climate refugia have stronger wind energy by 0.02 W/m^2^ SE 0.004 (<.0001 *p*‐value) and stronger tidal energy by 0.64 W/m^2^ SE 0.08 (<.0001 *p*‐value) than the rest of the GBR (Figure 2b,c). The relief from warming provided by tidal energy will persist into the future, but the locations and strength of wind energy are vulnerable to climate change.

### 2.2 Loss of climate refugia

**FIGURE 3:**
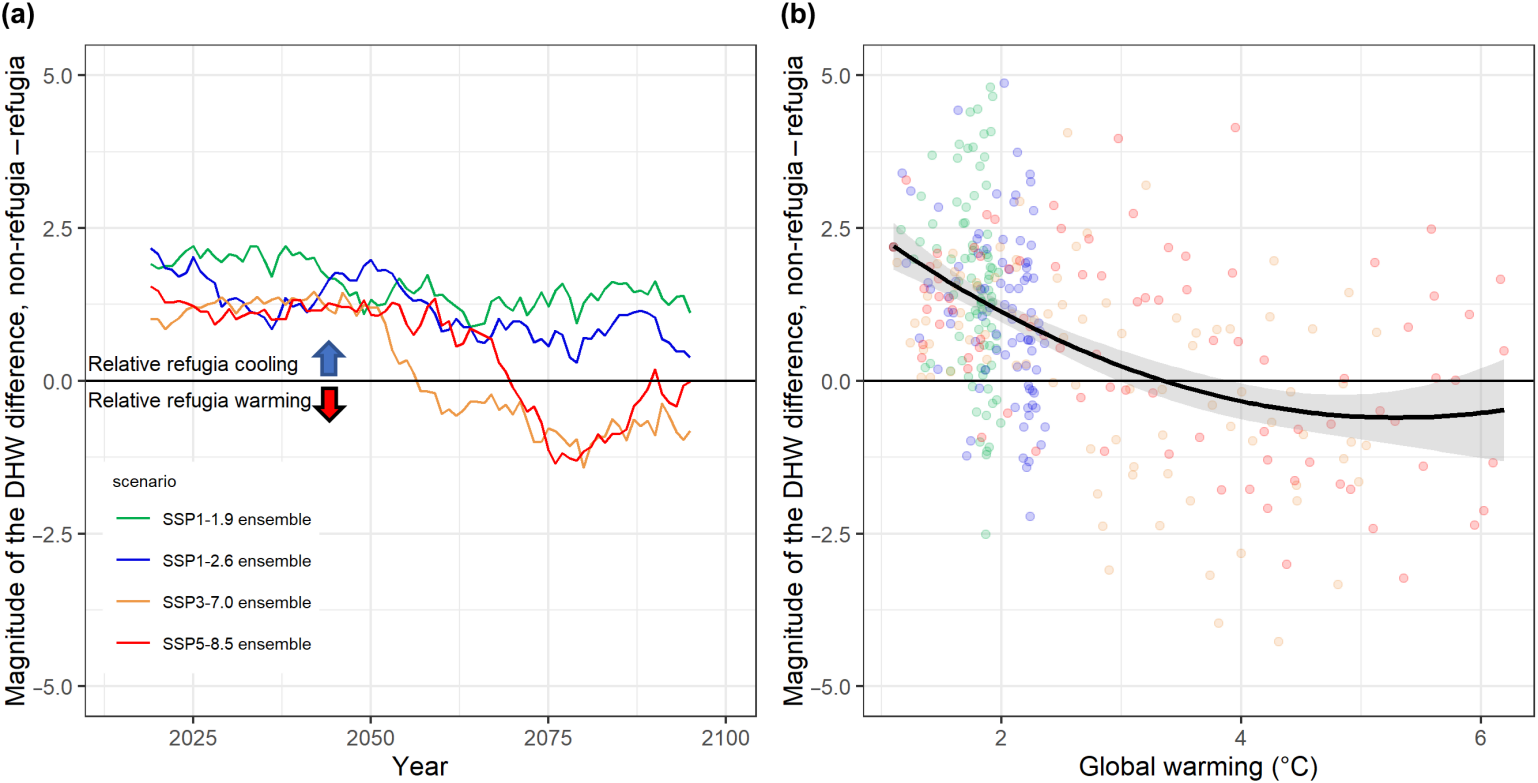
Refugia outlook under climate change scenarios. (a) Following a rolling mean of 11 years, DHW values in refugia locations were subtracted from the non‐refugia locations per model and then calculated as an ensemble mean difference per scenario, displaying the difference per year in DHW. (b) Differences prior to the rolling window were then plotted against global average temperatures relative to pre‐industrial time (1860–1880) from the corresponding climate model. A second‐degree polynomial was applied to all scenarios with shaded areas denoting standard error. DHW, degree heating week.

Under low emissions equating to <1.5°C or <2.0°C of warming by 2100 (SSP1‐1.9 and SSP1‐2.6), climatologically identified refugia typically persist in experiencing the lowest 20% of annual mean DHW values within any particular year, until at least the end of this century (i.e., ensemble mean refugia locations maintain lower DHW than non‐refugia, Figure 3a). In contrast, most refugia are lost (i.e., do not stay below the 20th percentile of annual DHWs) after the mid‐century under the two high emissions scenarios, SSP3‐7.0 and SSP5‐8.5 (Figure 3a). Irrespective of emissions scenario we find that refugia are typically lost after ~3° of globally averaged warming above preindustrial (1860–1880) levels (Figure 3b).

**FIGURE 4:**
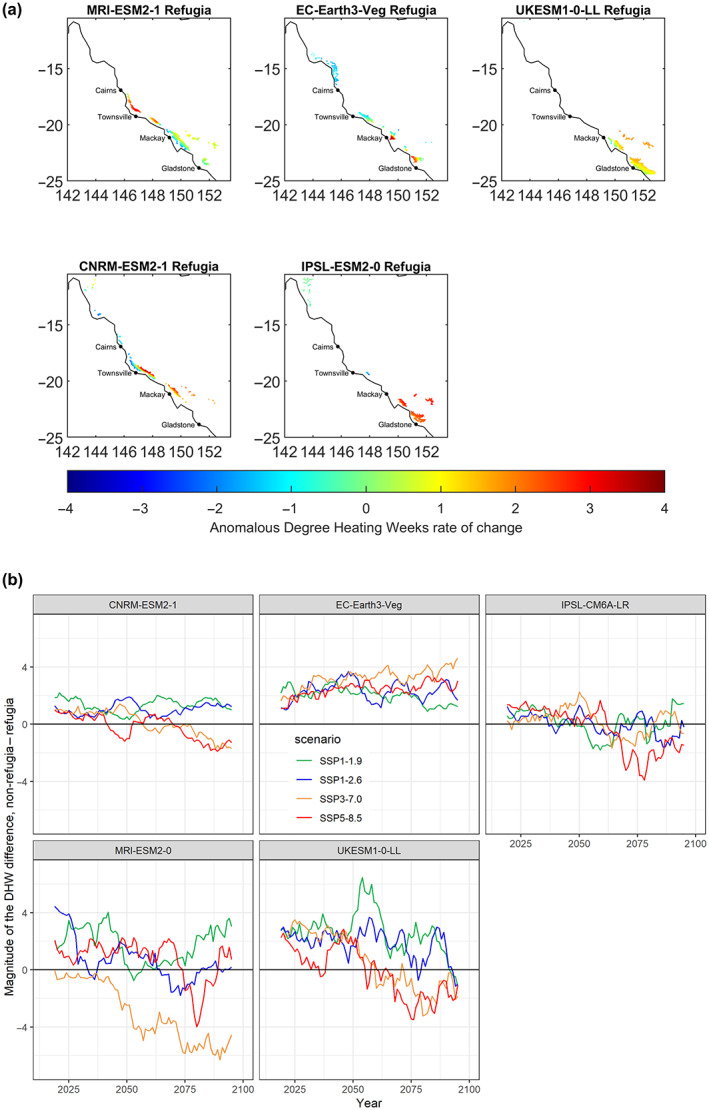
Refugia locations are shown per model to further identify spatial trends. (a) The rate of warming (slope) for refugia locations was calculated using DHW input values from the highest socioeconomic pathway, SSP5‐8.5, per model relative to the median value across the entire GBR grid, 142.0° W, 157.0° E, 30.0° S, 10.0° S at a 10 km horizontal resolution over depths of 4–50 m. The median value per year across the GBR grid was subtracted from each cell per year, then a least squares linear regression was fitted per cell across the entire time series, 2014–2100. Austral summer was placed in the middle of the calendar year when calculating the annual maximum temperature to avoid double counting. The blue colours indicate less warming relative to the median value across the GBR while red colours indicate the most relative warming. (b) Complementary to Figure 2 but displayed per model to highlight the difference in DHWs between refugia and non‐refugia per year in DHWs. DHW, degree heating week; GBR, Great Barrier Reef.

**FIGURE 5:**
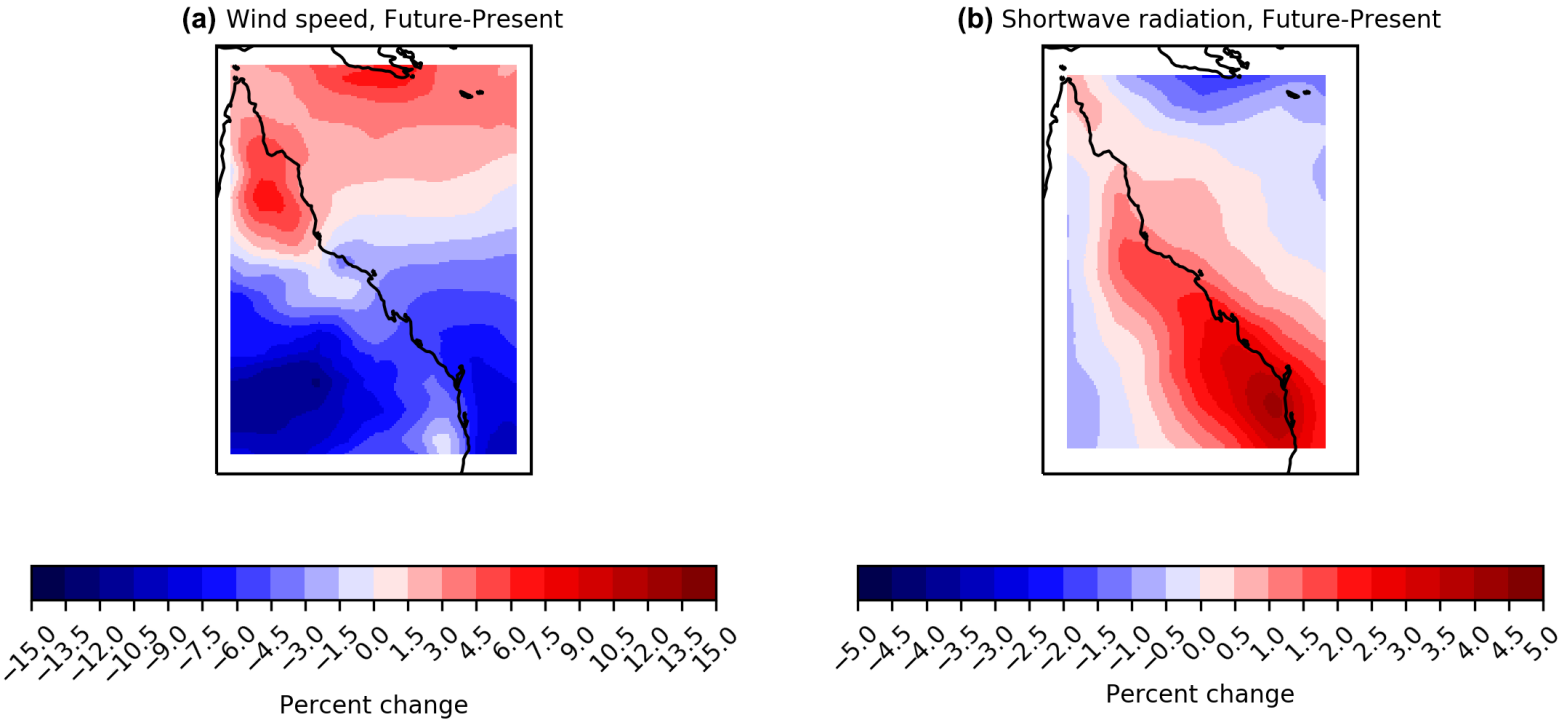
Twenty‐first century wind speed and shortwave radiation change. SSP5‐8.5 bleaching conditions were isolated within the (a) wind speed and (b) shortwave radiation variables displaying the percentage of change in present‐day (1999–2019) to future (2050–2100) conditions under SSP5‐8.5. Bleaching conditions are defined as austral summer months, December, January, February, March, calculated as austral summer years (i.e., July 31, 2050–August 1, 2051) with the median value across the GBR grid having an annual maximum DHW ≥2. DHW, degree heating week; GBR, Great Barrier Reef.

The rate of refugia loss varies across the reef with refugia in northern regions persisting longer than those in central and southern regions (Figure 4). CMIP6 projections suggest that a north–south dipole exists in wind‐speeds at times when bleaching is occurring (Figure 5a), such that wind speeds decline in southern areas, which reduce the mixing—and therefore cooling potential—of the water column. In contrast, wind speeds increase in the north (Figure 5a), elevating mixing potential. Moreover, the southern GBR is projected to experience a relative increase in shortwave radiation, which can both increase heating and potentially exacerbate the photosynthetic stress that causes bleaching (Enríquez et al., 2005; Skirving et al., 2017) (Figure 5b).

## 3 DISCUSSION

Global‐scale coral bleaching events are expected to occur more regularly as global average temperatures increase (Dixon et al., 2022; Frieler et al., 2013; Van Hooidonk et al., 2016). A recent SST based study using statistical downscaling by Dixon et al. (2022) projects a loss of nearly all (84.1% globally and 86% on the GBR) refugia under 1.5°C and a complete loss of the remaining refugia under 2°C. Differences in the locations of refugia in the Dixon study were hypothesized to be due to interannual and seasonal SST variability (Dixon et al., 2022).

The semi‐dynamic downscaling processes in this study enable a more sophisticated spatiotemporal analysis of climate projections over coral reefs. Accounting for tides and winds prove to be important variables when quantifying areas of climate refugia in the context of thermal exposure.

### 3.1 Atmospheric circulation driving changes to wind and shortwave radiation

The modelled change in shortwave radiation (Figure 5b), linked to increased bleaching in the Southern GBR, is potentially driven by a projected weakening in the Hadley circulation influencing the descending branch over the GBR known as the Subtropical Ridge (Timbal & Drosdowsky, 2013). As the planet warms, climate models project an expansion of the Hadley circulation poleward (Dey et al., 2019; Frierson et al., 2007; Hu et al., 2013; Lu et al., 2007; Seidel et al., 2008) causing an intensification and poleward shift of the Subtropical Ridge (Dey et al., 2019; Grose et al., 2015; Kent et al., 2013). The latitudinal dipole in changing wind speeds projected into the future (Figure 5a) can potentially be explained by an increase in the summer monsoon intensity during bleaching conditions (Brown et al., 2016; Dey et al., 2019).

### 3.2 Climate model variability

The use of an ensemble of climate models, rather than a single model, allows us to explore the most likely outcome, i.e., the multi‐model mean (IPCC, 2021), but also alternative potential outcomes. Downscaling of one of the five models—EC‐Earth (Döscher et al., 2021)—gave more optimistic projections than the others. Using this model, relative thermal refugia persist under all four climate scenarios (Figure 4b). Our analysis using EC‐Earth predicts more refugia in the north than we see with other models (Figure 4a). This climatological distribution of refugia, combined with the northerly bias seen in refugia persistence, contributes to the anomalous refugia persistence identified from EC‐Earth. Further analysis of individual models can be found in the Supplementary Material.

### 3.3 Downscaling limitations

Large mesoscale processes will result in lateral advection not simulated within the downscaling presented here. Lateral advection could occur through eddies, western boundary currents, the Hiri and the East Australian currents. Glynn (1996) hypothesized that vigorous circulation (for example in upwelling centres, oceanic banks, island shores) may provide corals with a refuge from warming ocean temperatures (Baird et al., 2018; Glynn, 1996). The South Equatorial Current reaches the Australian continent between latitude 14°S and 18°S (Andrews & Clegg, 1989; Burrage, 1993; Church, 1987) when the East Australian Current then flows southward and the Hiri Current flows northward. The location of bifurcation varies seasonally and inter‐annually and these currents mostly influence waters on the outer shelf (Burrage, 1993; Wolanski & Spagnol, 2000). This location of bifurcation marks the division of the warm tropical and cool subtropical gyres. The impact of these processes on the downscaling presented here will only be felt through the imposed atmospheric forcing. Despite these limitations, the dominance of atmospheric forcing in driving shallow water extreme events, and importance of tidal mixing in moderating these events (Halloran et al., 2021), combined with the potential offered by a simplified, and therefore computationally efficient, shelf sea model to undertake multi‐model and model‐scenario downscaling, make such an approach very valuable.

### 3.4 Climate trajectory uncertainty for corals

Even under the lowest emissions trajectories we still face a committed warming. The frequency of severe coral bleaching events (when DHW >8) is expected to be five events per decade under SSP1‐2.6 and three events per decade under SSP1‐1.9 surrounding 2060 (McWhorter et al., 2021). The increasing frequency and magnitude of warming events into the future make corals extremely vulnerable to mortality from climate change (Bozec et al., 2020; Frieler et al., 2013; Hoegh‐Guldberg, 1999; Hughes et al., 2017; Hughes, Anderson, et al., 2018; King et al., 2017; Van Hooidonk et al., 2016). One widespread warming event, or a succession of warming events could have severe and irreversible consequences eliminating any concept of a refugia. Under low emissions trajectories, the locations of refugia show an average trend of less DHW than the rest of the GBR, which means these locations are slightly less vulnerable in terms of magnitude, yet not necessarily in terms of frequency. Even during more frequent events, if these refugia contain a lower magnitude of stress than the rest of the GBR, such locations could become increasingly valuable to the entire reef system as sustained refugia or areas to facilitate recovery (Bozec et al., 2020; Hock et al., 2017).

Global policy decisions that would result in high emissions trajectories will likely be at the cost of the identified refugia in this study. Low emissions trajectories offer a less impacted future with a greater opportunity for recovery and survival. If these refugia maintain their less impacted status, they could potentially provide larvae to hydrodynamically connected reefs, facilitating the recovery process after bleaching events (Bode et al., 2018; Cheung et al., 2021; Hock et al., 2017). The identified persistent refugia could be targets for management interventions, i.e., further protection such as limitations on overfishing of herbivores (Bozec et al., 2020; Doropoulos et al., 2016; Graham et al., 2015; Hughes et al., 2007; Mumby et al., 2016), the management of nutrient pollution and invasive species control (Brodie et al., 2012). Indeed, the incorporation of ‘climate refugia’ into management does not preclude the importance of managing other processes that can impact reef trajectories, including water quality and fisheries impacts. Rather, there may be significant benefits in directing some of those standard management practices towards refugia, particularly where they help the robustness of coral populations over time.

### 3.5 Coral adaptation and recovery

Climate refugia are likely to support a high abundance of coral colonies relative to non‐refugia and may act as important sources for larval export (Cheung et al., 2021). However, the role of such areas for coral adaptation are now the subject of intensive research (McManus et al., 2021; Walsworth et al., 2019) and the outcome remains unclear. On the one‐hand, they may have a positive role in bolstering population size and turnover in other sections of the reef. The maintenance of higher population size will contribute to ecosystem functioning, such as calcification and reef building (Wolfe et al., 2020) and possibly to larger effective (genetic) population size. However, a lower selection pressure on thermal tolerance traits in stress refugia may lead to an accumulation of corals which are maladapted to non‐refugia conditions which would potentially slow the rate of adaptation in stressed areas downstream (McManus et al., 2021). Nonetheless, both climate refugia and areas of intense heat stress have roles to play in designing future conservation strategies for the GBR. Our results suggest that such investments in planning are worthwhile because refugia can continue to exist for at least 30 years and potentially longer if global emissions can be contained.

## 4 MATERIALS AND METHODS

### 4.1 Downscaling model data

The S2P3‐R v2.0 semi‐dynamic (Halloran et al., 2021) downscaling method is driven by fully coupled GCM variables of surface atmosphere air temperature, winds, air pressure, humidity, and net longwave and shortwave radiation with high resolution bathymetry (Beaman, 2010) and tidal components to calculate water column properties. The domain of the model spans 142.0° W, 157.0° E, 30.0° S, 10.0° S with a 10 km horizontal resolution and 2 m vertical resolution. The model was run in water depths from 4 to 50 m. The S2P3‐R v2.0 model is driven with surface level atmospheric data from CMIP6 models, GCMs, MRI‐ESM2‐0, EC‐Earth3‐Veg, UKESM1‐0‐LL, CNRM‐ESM2‐1 and IPSL‐ESM2‐0.

Initially, a tidal slope is calculated from M2, S2, N2, O1 and K1 tidal ellipses to then calculate the water's velocity 1 m above the seabed. Water velocity interacts with a prescribed standard bottom drag coefficient (Sharples et al., 2006). Wind velocity is calculated with respect to tides and air pressure, as it interacts with a surface drag coefficient (Smith & Banke, 1975). Profiles of vertical eddy viscosity and diffusivity are calculated in a turbulence closure scheme as functions of current shear and vertical density (Canuto et al., 2001) then used with the surface and bottom stress calculations. Density is described in the model only as a function of temperature. SST data from 1950 to 2100 were output daily. Further information on validation and physical components of the model are in Halloran et al. (2021).

### 4.2 Shared socioeconomic pathways

There are five SSPs for various possible socio‐economic developments. The pathways include sustainable development, inequality, regional conflict, fossil fuel‐based development, and middle‐of‐the‐road development. While consistent with the literature, there is a wide range of uncertainty surrounding economic and demographic projections. Within this study, we focused on three SSP trajectories (SSP1, SSP3, SSP5) (Riahi et al., 2017) and four emissions trajectories (SSP1‐1.9, SSP1‐2.6, SSP3‐7.0, SSP5‐8.5) (Riahi et al., 2017). The last numbers (1.9, 2.6, 7.0, and 8.5) refer to the peak radiative forcing (W/m^2^). SSP1 refers to the most sustainable future, involving low material growth and lower resource and energy intensity. As a result of development goals being more focused on the global commons investing in education, health and economic growth emphasizing human well‐being, inequality is reduced across countries. SSP3 focuses on ‘regional rivalry’, a rise in nationalism, a scenario where regional competitiveness and conflict drives countries to focus on their own energy and food security goals. Additionally, investments in education and technology decline, inequalities worsen, and economic development is slow. Population growth in this scenario is high in developing countries and low in industrialized countries. Also, the international community does not prioritize environmental issues in SSP3. SSP5 is a world based on fossil fuel development, energy intensive lifestyles which grow the global economy and population. Competitive markets drive technology, innovation, and human capital towards sustainable development. Population peaks and then declines; local environmental problems are successfully managed and solutions such as geo‐engineering maybe be included to manage social and ecological systems (Riahi et al., 2017). While SSP1‐1.9 and SSP1‐2.6 are within the same SSP category (SSP1), they contain different radiative forcing pathways. SSP1‐1.9 was designed to limit warming to 1.5°C by the end of the century. This scenario uniquely contains the application of technology which extracts large amounts of CO_2_ out of the atmosphere resulting in net negative emissions in the second half of the 21st century (O'Neill et al., 2016).

### 4.3 Coral stress metrics

NOAA Coral Reef Watch's operational suite of coral heat stress products have been used by the global coral reef community for more than two decades (www.coralreefwatch.noaa.gov). The DHW product is by far the most used metric used by reef managers and scientists to monitor coral bleaching related heat stress. This study uses the exact methodology used by NOAA Coral Reef Watch and described in Skirving et al. (2020) to derive coral stress metrics. Following is a brief description of the methodology. More detail and the rational for the methodology can be found in Skirving et al. (2020).

A monthly mean climatology was initially created for each grid point. For each grid point, monthly mean values (12) were calculated from 1985 to 2012 and linearly adjusted to 1988.2857 to be consistent with the original NOAA Coral Reef Watch Maximum Monthly Mean (MMM) climatology. The original NOAA Coral Reef Watch climatology, that is, 1985–1990 and 1993 was adjusted to account for missing years due to aerosol contamination from the Mt. Pinatubo eruption. Although modern satellite data now account for the missing years, the climatology remains adjusted to match the original 7‐year climatology.

Daily SST values in each month were averaged to produce 12 mean SST values for each of the 28 climatology years (1985–2012). Then a least squares linear regression was applied to each month corresponding to the temperature value in 1988.2857. For example, to derive the January value, the 28 January averages (*Y*‐values) were regressed against the years (*X*‐values), and the temperature value when *X* = 1988.2857 was assigned as the monthly mean value. This was repeated for each of the 12 months and at each pixel location until each pixel had a set of 12 monthly mean values which represents the monthly mean climatology for 1988.2857. The maximum of these 12 monthly means is the Maximum Monthly Mean, called MMM (Skirving et al., 2020).

The next step is to use the MMM to create values for a warm SST anomaly, called a ‘HotSpot’ (Skirving et al., 2020). Daily SST values are first subtracted from the MMM climatology. Then, all negative values are set to zero to select only warm anomalies, therefore, ‘HotSpot’ ≥0. The DHW calculation is then a daily summation over an 84‐day running window of the ‘HotSpot’ values. Additionally, the DHW calculation only selects ‘HotSpot’ values greater than or equal to 1. Thermal stress amongst corals has been considered to begin at MMM + 1°C (Skirving et al., 2020).

Further, the maximum DHW was used per grid cell per year. For time series calculations, the median DHW value was then taken annually across the spatial domain for each model in each scenario. Then, the median DHW values were further averaged using all models within each scenario resulting in an ensemble median per scenario.

### 4.4 Refugia calculation

Austral summer years (i.e., July 31, 1999–August 1, 2000) were used to calculate the annual maximum DHW, avoiding double counting when using calendar years (Skirving et al., 2019). Refugia locations were found by calculating the average DHW per cell from 1999 to 2019, then, calculating the 20th percentile value across the GBR grid from the downscaled output [142.0° W, 157.0° E, 30.0° S, 10.0° S with a 10 km horizontal resolution from 4 to 50 m depths, within the Great Barrier Reef Marine Park Authority (GBRMPA) boundary (GBRMPA, 2009)]. All cells that were less than or equal to the 20th percentile value were kept as ‘refugia’ locations. ‘Non‐refugia’ locations were the remaining cells. This method is consistent with the literature (Cheung et al., 2021; Hock et al., 2017).

### 4.5 Global warming metrics

Global average temperatures were extracted per climate model using the historical data surface air temperature, the ‘tas’ variable. Global average temperatures were calculated relative to pre‐industrial time (1860–1880).

The relationship between global warming and the magnitude of the DHW difference, non‐refugia–refugia was modelled using a generalised additive model with a scaled *t*‐distribution and with a spline with three knots. Models were fit using the bam function in the ‘mgcv’ package in R and using the scaled to family with a logarithmic scale (link = “scat”). Significant differences were tested using Tukey adjusted pairwise comparisons using the emmeans function in the ‘emmeans’ package (Lenth et al., 2018).

### 4.6 Present‐day potential energy anomalies; wind and tidal energy flux metrics

Atmospheric reanalysis product ERA5 was downscaled using S2P3‐R v2.0 to provide a link between observational outputs and climate model outputs. Energy flux from winds and tides within the water column are outputs of the S2P3‐R v2.0 downscaling process. The turbulent power generated by tides is calculated using the bottom drag coefficient, *k*_b_ = 0.003, the density of seawater, *ρ*, and the amplitude of the tidal current, *u*_0_.

$$P_{\text{tide}}=\frac{4k_{b}\rho u_{0}^{3}}{3\pi }$$

Wind mixing at the sea surface is derived from *δ* as wind mixing. Further, *k*_s_ represents 6.4 × 10^−5^ of the drag coefficient multiplied by the slippage factor. *w* represents wind speed, *h* represents total depth, and *ρ*_a_ represents air density.

$$\frac{{\partial \phi }_{\text{wind}}}{\partial t}=-\delta k_{s}\rho_{a}\frac{w^{3}}{h}$$

The wind and tidal energy flux were calculated using the average conditions during austral summer months, December, January, February, March, over refugia and non‐refugia locations. For the wind energy flux analysis, only mass coral bleaching years were used from 1999 to 2019 (2002, 2016, 2017) (Hughes et al., 2017; Hughes, Kerry, et al., 2018). Refugia locations for this analysis are defined as locations where the lowest 20th percentile DHW values from the downscaled ERA5 dataset are observed and locations where 2–5 models agree with ERA5 refugia location. Non‐refugia locations are all other locations, or where 0–1 models agree.

Additive mixed effect models were used to explore differences between refugia and non‐refugia locations using the ‘bam’ function (Wood, 2004) in R version 4.1.1 (Pinheiro et al., 2021) where longitude and latitude were included as a random effect to account for the spatial correlation of the data. Pairwise comparisons were determined using the ‘pairs’ function (Lenth, 2021) in R version 4.1.1 (Pinheiro et al., 2021).

### 4.7 Rate of warming relative to GBR median DHW calculations

Annual maximum DHW inputs from SSP5‐8.5 were used to analyse the rate of warming per model. The non‐refugia GBR median was calculated per year and then subtracted from each refugia grid cell in that year. Following this step, a linear regression was fitted to the timeseries of each cell, resulting in a metric of relative warming to the non‐refugia locations, or slope.

### 4.8 Bleaching conditions

Bleaching conditions were defined by isolating austral summer months December, January, February, and March, and then selecting the years where the median DHW value across the GBR was ≥2. It was common for most years following 2050 among all models to fall under ‘bleaching conditions’ in the highest scenario, SSP5‐8.5.

### 4.9 Wind speed and shortwave radiation during bleaching conditions

The five models used in the downscaling from the highest emission scenario (SSP5‐8.5) were used to analyse the wind speed and shortwave radiation conditions during present‐day and future bleaching conditions. Present day years were isolated to 1999–2019 and future years were 2020–2100. It was common that most years following 2050 among all models fall under bleaching conditions in the highest scenario SSP5‐8.5.

## AUTHOR CONTRIBUTIONS

**Jennifer K. McWhorter, Paul R. Halloran, Peter J. Mumby:** Conceptualization. **Jennifer K. McWhorter, Paul R. Halloran, Peter J. Mumby, George Roff, William J. Skirving:** Methodology. **Jennifer K. McWhorter, Paul R. Halloran, Peter J. Mumby, George Roff:** Investigation. **Jennifer K. McWhorter, Paul R. Halloran, Peter J. Mumby, George Roff:** Visualisation. **Paul R. Halloran, Peter J. Mumby, George Roff:** Supervision. **Jennifer K. McWhorter, Paul R. Halloran, Peter J. Mumby:** Writing – original draft. **Jennifer K. McWhorter, Paul R. Halloran, Peter J. Mumby, George Roff, William J. Skirving:** Writing – review and editing.

## FUNDING INFORMATION

Financial support for this study was provided by the QUEX Institute, a University of Exeter and University of Queensland Partnership. PRH, WJS, and PJM were supported by the UK Research and Innovation grant NE/V00865X/1 and ARC grants. WJS was supported by NOAA grant NA19NES4320002 (Cooperative Institute for Satellite Earth System Studies) at the University of Maryland/ESSIC. The scientific results and conclusions, as well as any views or opinions expressed herein, are those of the author(s) and do not necessarily reflect the views of NOAA or the Department of Commerce.

## CONFLICT OF INTEREST

There are no potential conflicts of interest.

## Supporting information


Figure S1
Figure S2
Figure S3
Figure S4

## Data Availability

These data are available through Zenodo as the data were used in a previous paper (McWhorter et al., 2021), https://zenodo.org/record/5534875#.YnvfQOjMKUm. The code in this study is available by request.
