# Supplementary material for: Climate refugia on the Great Barrier Reef fail when global warming exceeds 3°C
Source: Glob Chang Biol. 2022 Aug 2;28(19):5768–80. doi: 10.1111/gcb.16323 (PMC9541460; doi:10.1111/gcb.16323)
Supplement: Supplementary file 1 — Figure S1 Figure S2 Figure S3 Figure S4 [file GCB-28-5768-s001.docx]

**Supplementary Material**


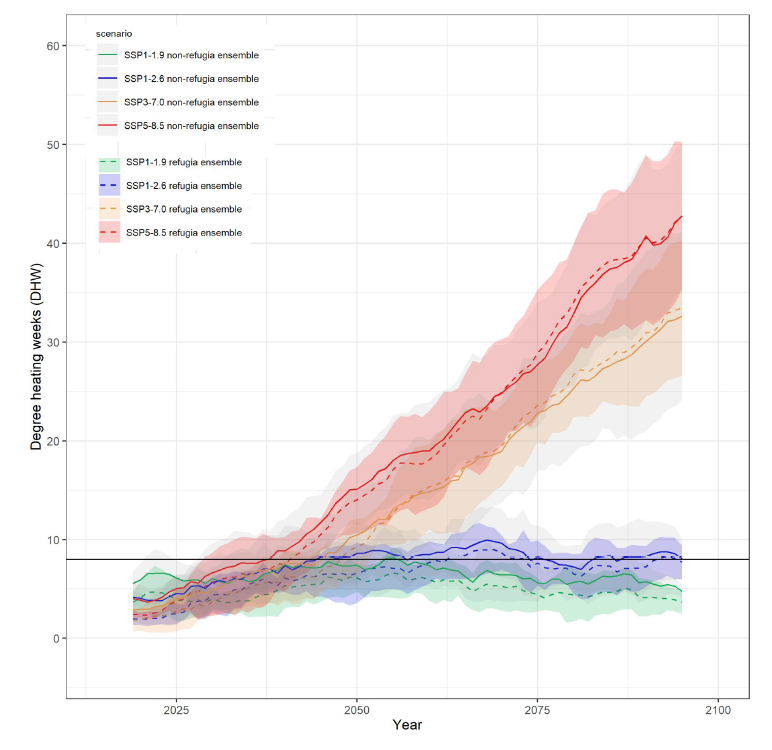


**Supplementary Figure 1.** Metrics of coral stress were extracted across the Great Barrier Reef for four socioeconomic pathways and an ensemble of five climate models for non-refugia and refugia locations. Coral stress metrics disaggregate magnitude as Degree Heating Weeks (DHW) shown using a rolling window of 11 years. The multi-model ensemble is made up of MRI-ESM2-0, EC-Earth3-Veg, UKESM1-0-LL, CNRM-ESM2-1, and IPSL-ESM2-0. Shaded areas denote the standard deviation for each scenario averaged across models. The horizontal black line in 1A marks 8 DHW, a metric of coral stress that often leads to mortality.


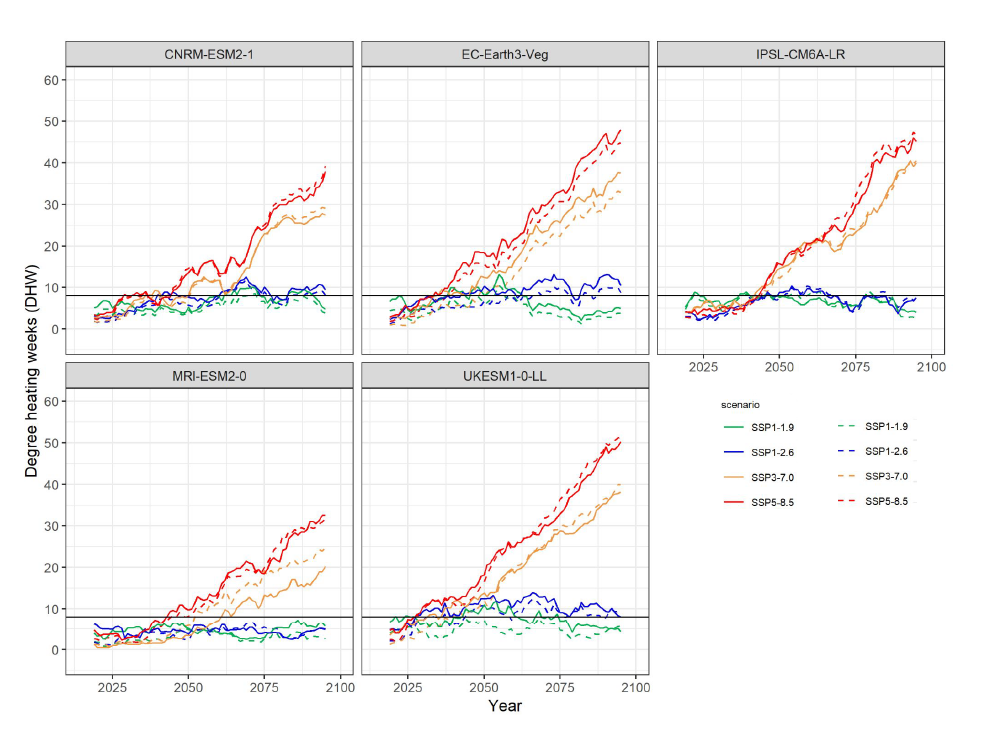


**Supplementary Figure 2.** Metrics of coral stress were extracted across the Great Barrier Reef for each of the five climate models with four socioeconomic pathways for non-refugia and refugia locations. Coral stress metrics disaggregate magnitude as Degree Heating Weeks (DHW) shown using a rolling window of 11 years. The horizontal black line marks 8 DHW, a metric of coral stress that often leads to mortality.

(a)


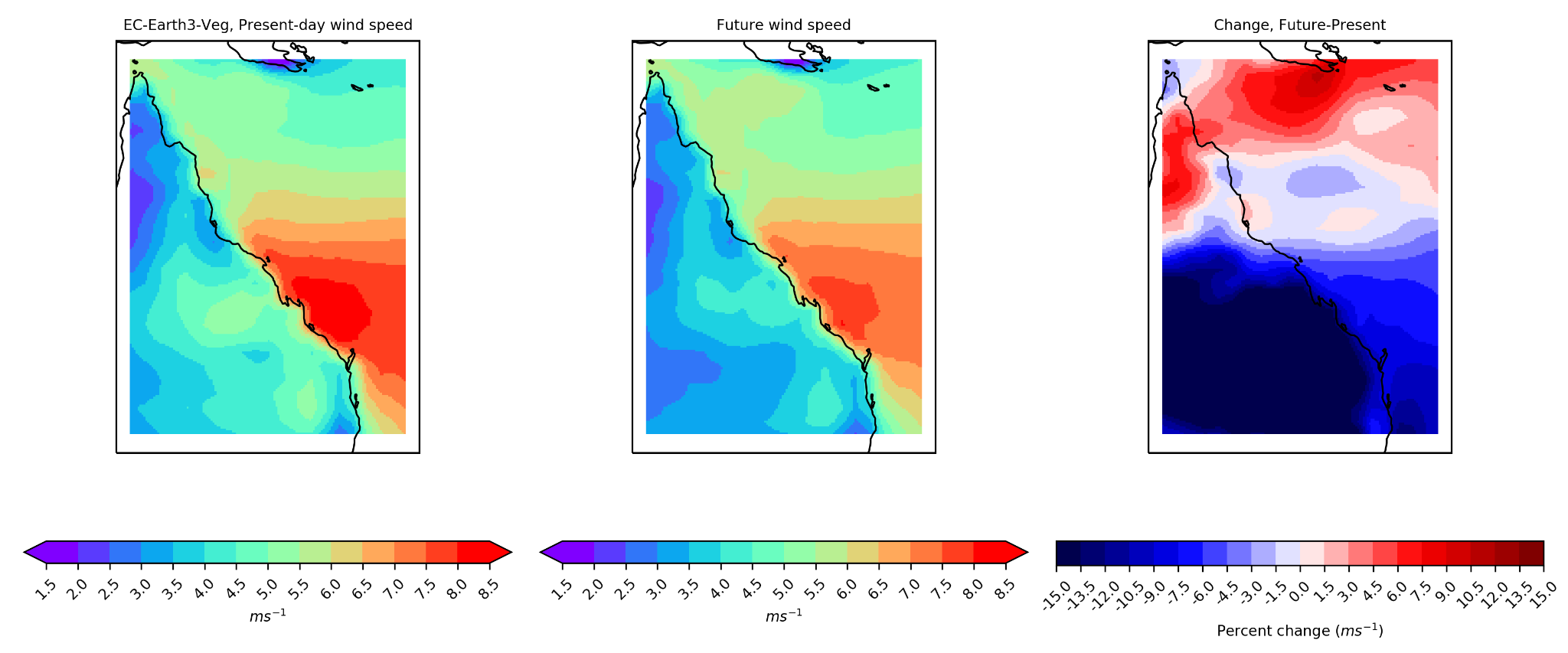


(b)


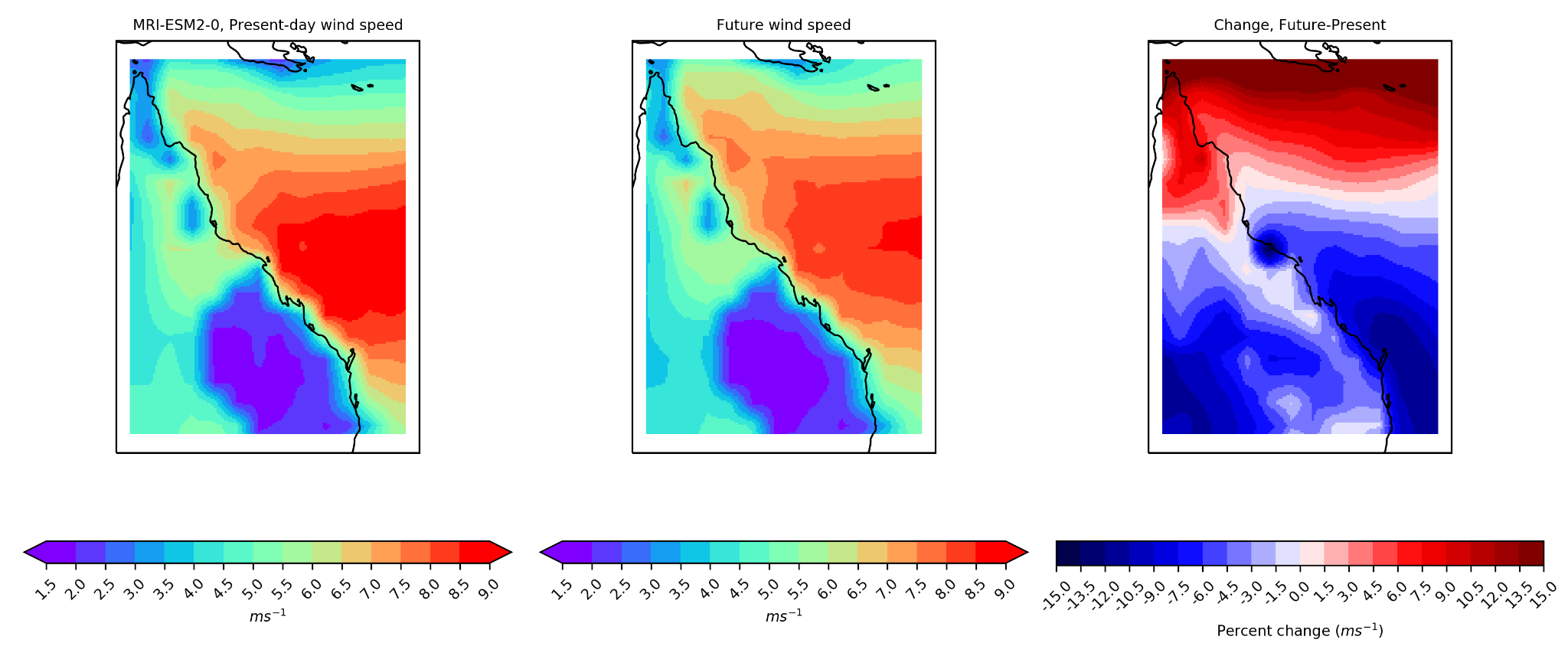


(c)


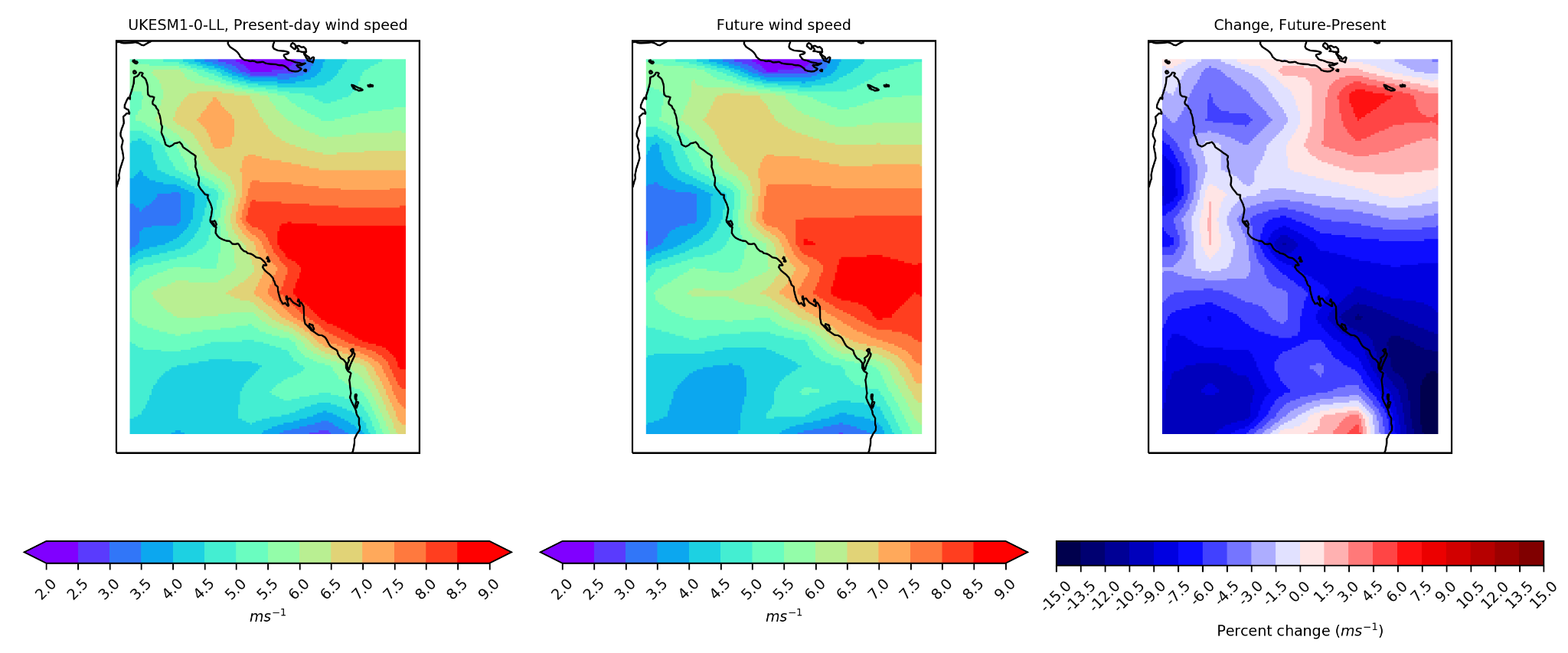


(d)


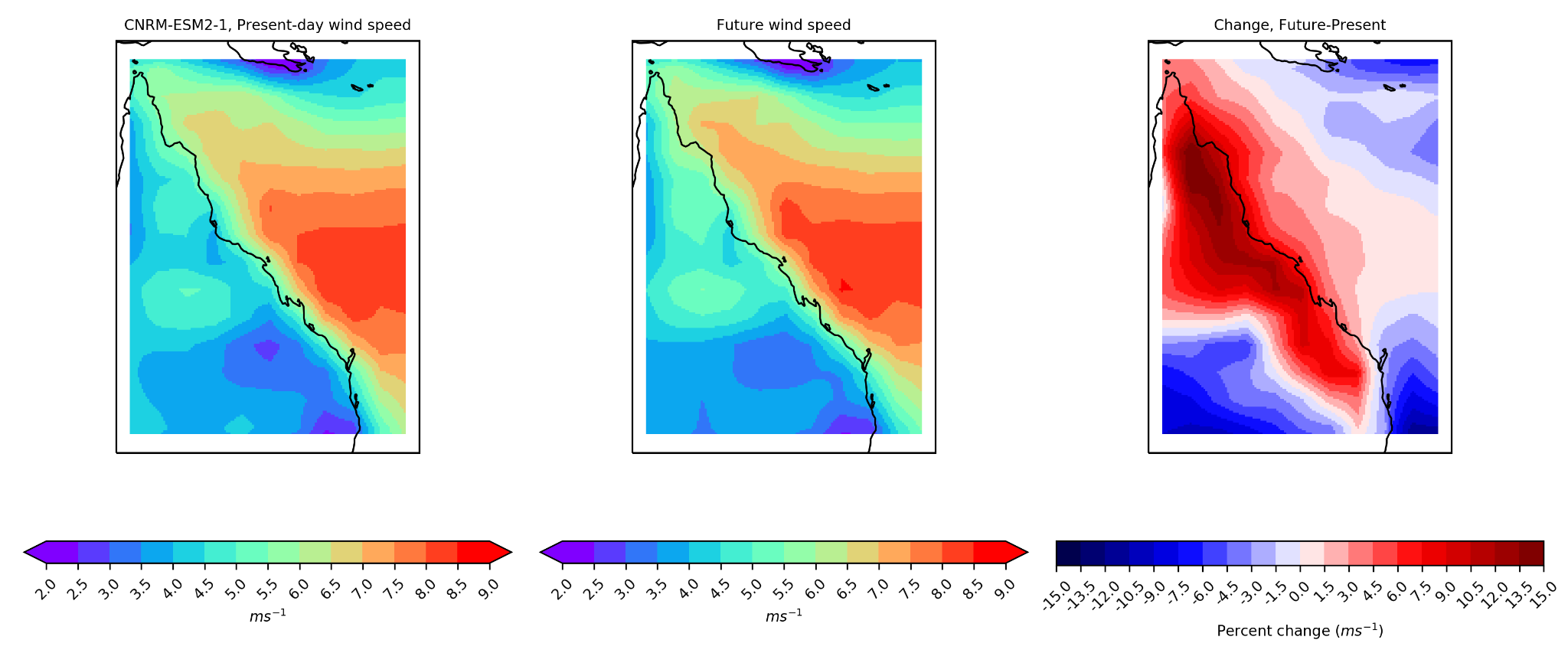


(e)


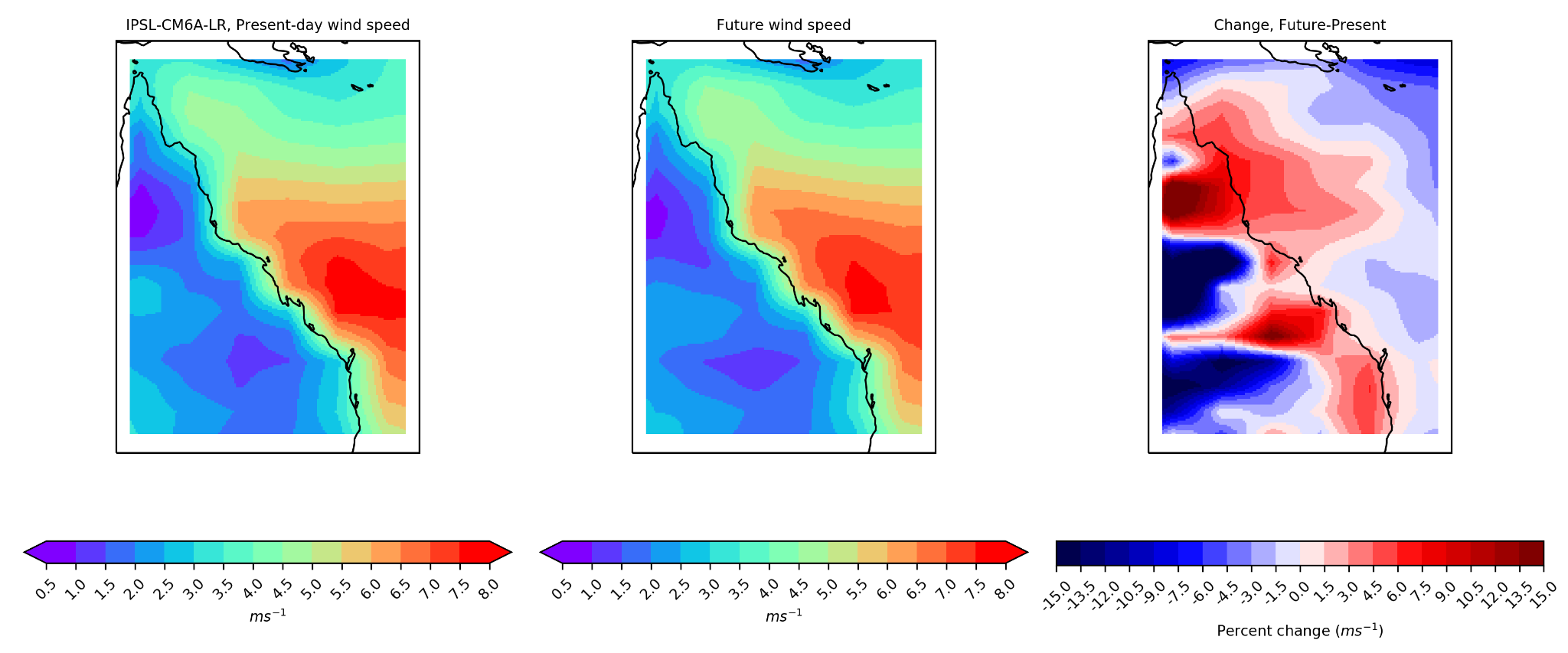


**Supplementary Figure 3.** Wind speed is shown under SSP5-8.5 for present-day, future, and the difference in wind speed during bleaching conditions per model (a) EC-Earth3-Veg, (b) MRI-ESM2-0, (c) UKESM1-0-LL, (d) CNRM-ESM2-1, and (e) IPSL-ESM2-0. Bleaching conditions are defined as austral summer months, December, January, February, March, calculated as austral summer years (i.e., July 31, 2050 – August 1, 2051) with the annual maximum DHW across the Great Barrier Reef Marine Park Authority boundary having a median value of DHW ≥ 2.

(a)


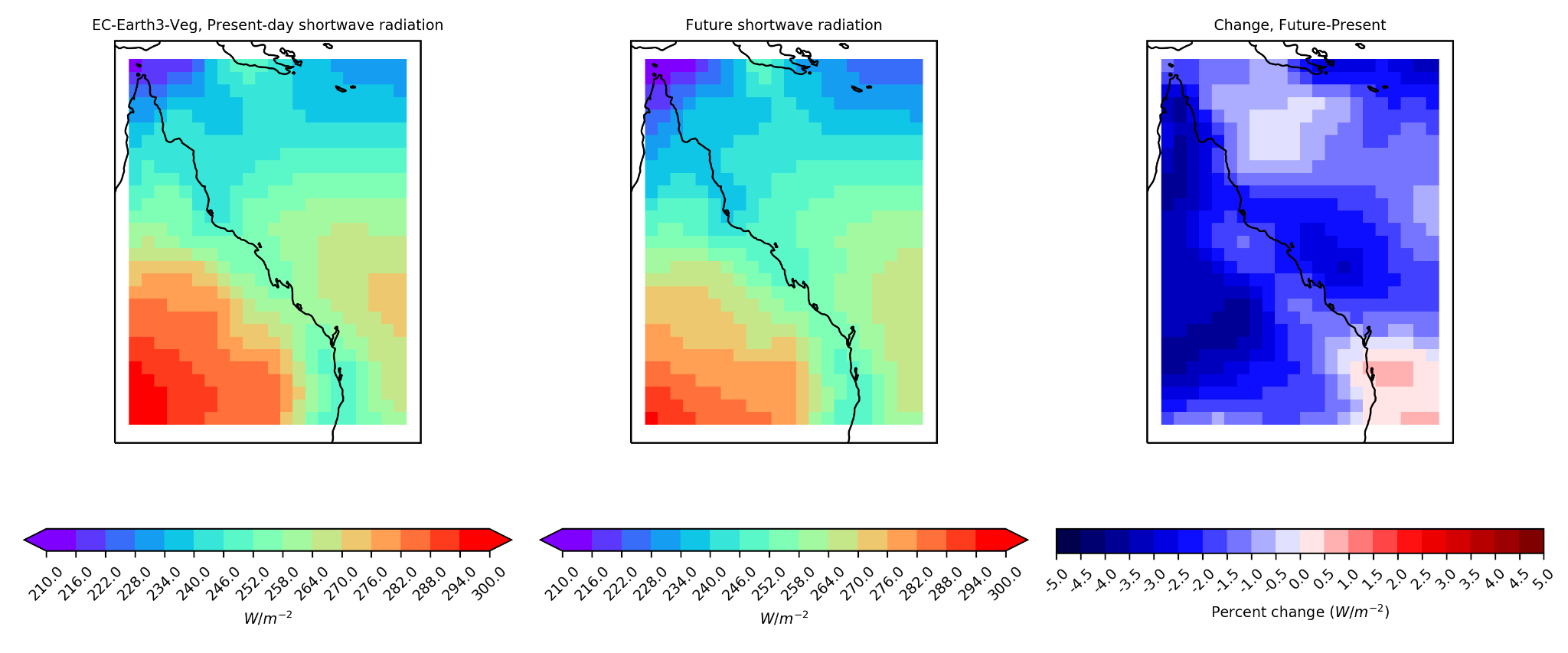


(b)


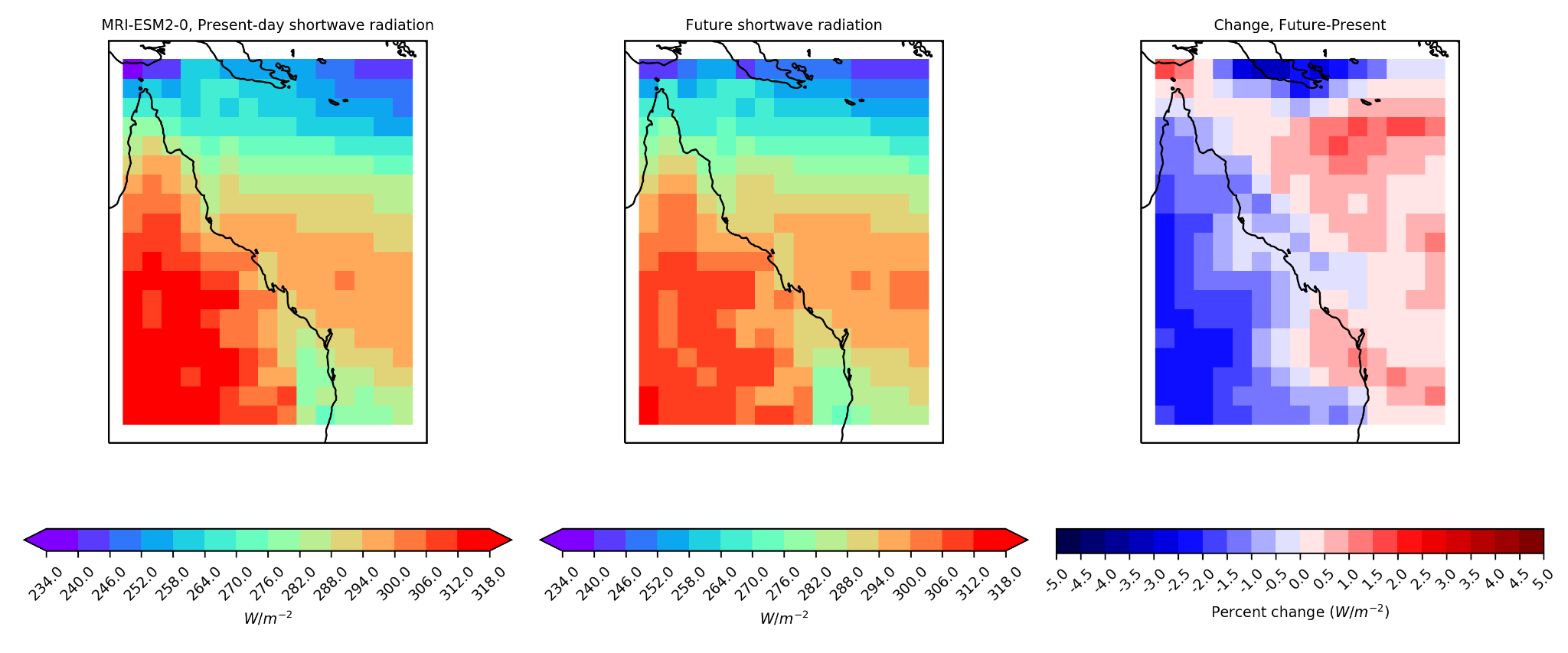


(c)


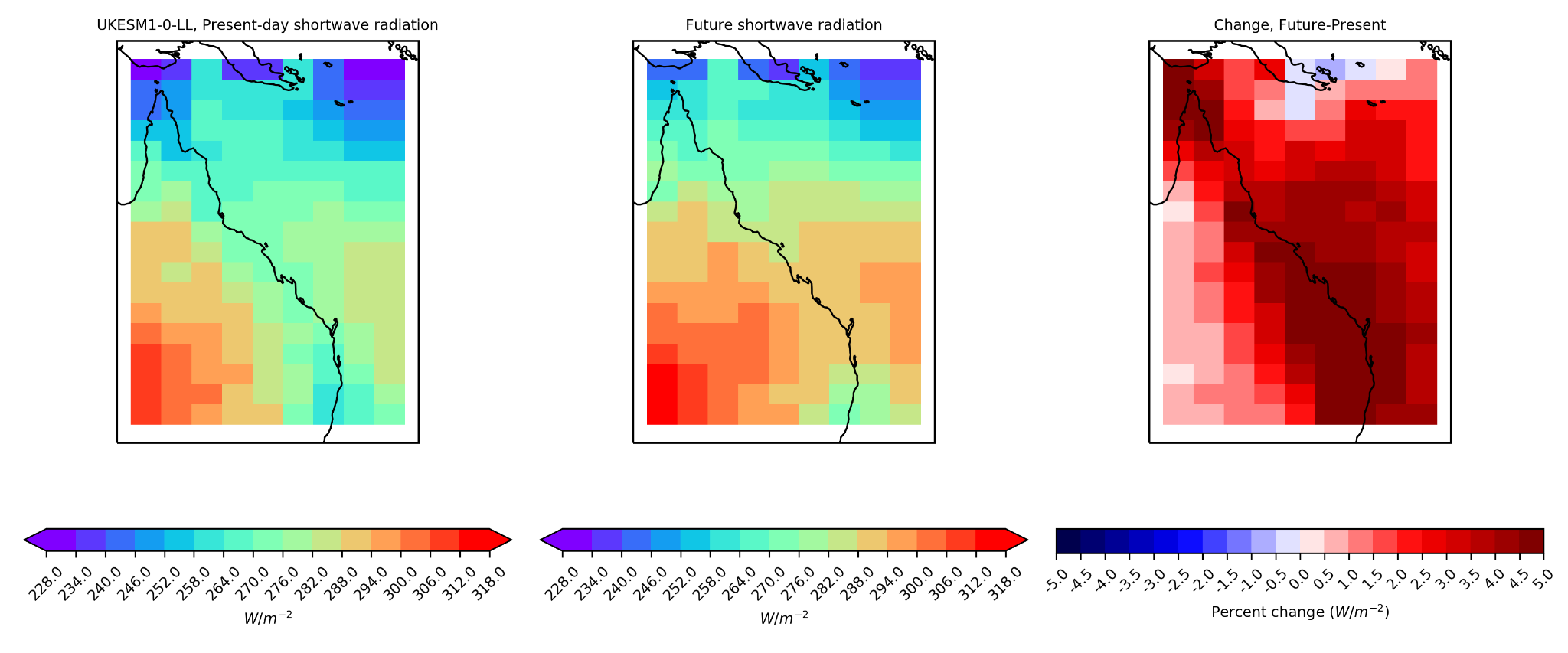


(d)


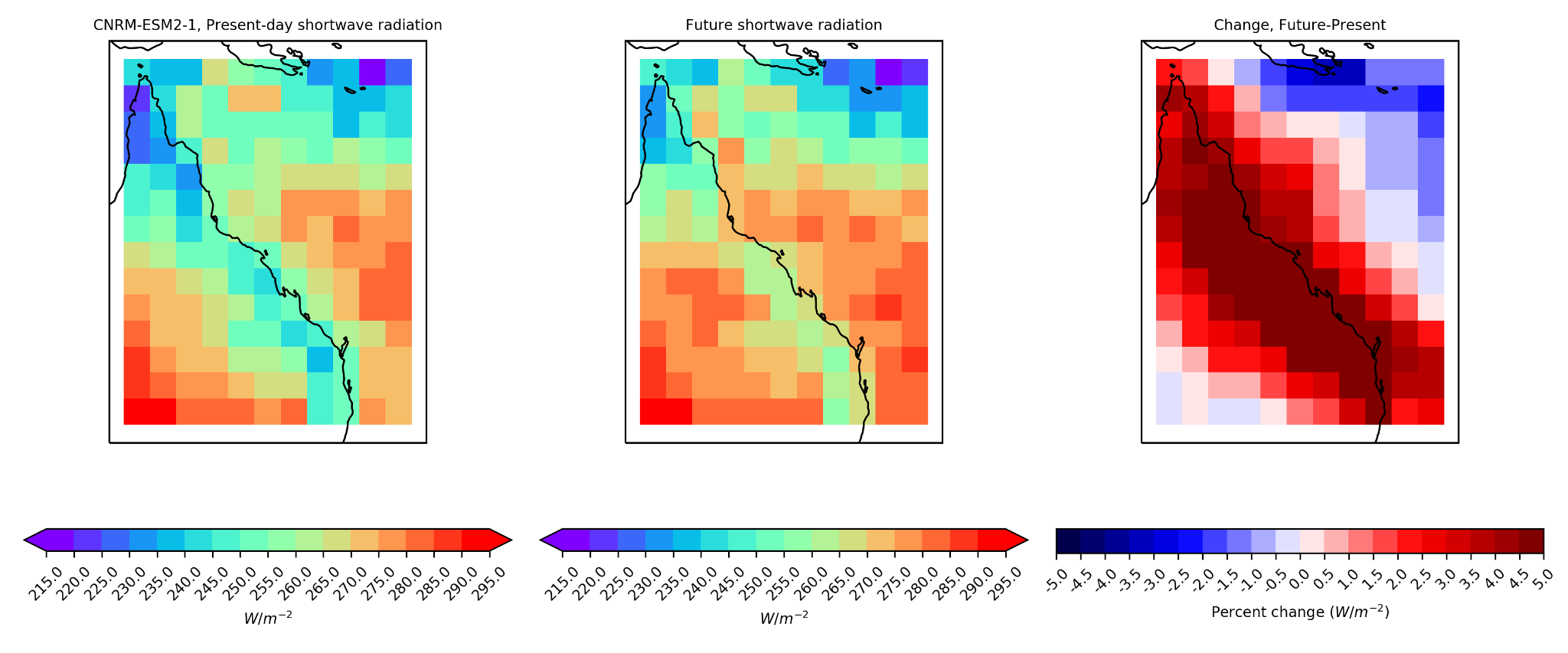


(e)


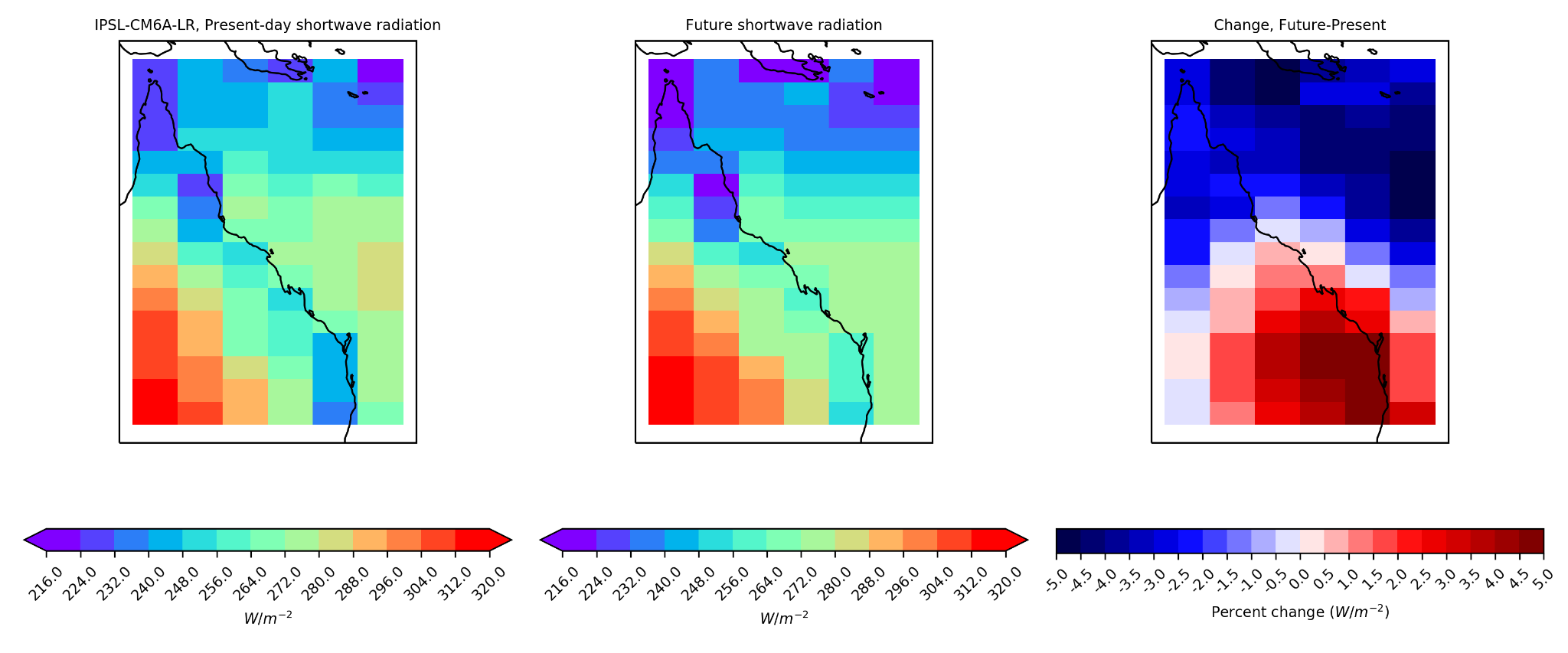


**Supplementary Figure 4.** Shortwave radiation is shown under SSP5-8.5 for present-day, future, and the difference in shortwave radiation during bleaching conditions per model (a) EC-Earth3-Veg, (b) MRI-ESM2-0, (c) UKESM1-0-LL, (d) CNRM-ESM2-1, and (e) IPSL-ESM2-0. Bleaching conditions are defined as austral summer months, December, January, February, March, calculated as austral summer years (i.e., July 31, 2050, – August 1, 2051) with the annual maximum DHW across the Great Barrier Reef Marine Park Authority boundary having a median value of DHW ≥2.
